# Persistence of *E. coli* in Streambed Sediment Contaminated with Faeces from Dairy Cows, Geese, and Deer: Legacy Risks to Environment and Health

**DOI:** 10.3390/ijerph20075375

**Published:** 2023-04-03

**Authors:** Emmanuel O. Afolabi, Richard S. Quilliam, David M. Oliver

**Affiliations:** Biological & Environmental Sciences, Faculty of Natural Sciences, University of Stirling, Stirling FK9 4LA, UK

**Keywords:** die-off, faecal indicator organism, mesocosm, river pollution, water quality, wildlife

## Abstract

Legacy stores of faecal pollution in streambed sediments can result in delayed impacts on environmental quality and human health if resuspended into the overlying water column. Different catchment sources of faecal pollution can contribute to a legacy store of microbial pollutants, with size of stores influenced by microbial die-off and faecal accrual rates in the streambed. The aim of this study was to use a mesocosm experiment to characterise the persistence of *E. coli* derived from faeces of dairy cows, deer, and geese once introduced to streambed sediment under different temperature regimes. The settling rate of solid constituents of faecal material into streambed sediment once delivered into an aquatic environment was also quantified. The persistence patterns of *E. coli* in streambed sediment were found to vary as a function of faecal source and temperature; die-off of *E. coli* in sediment contaminated with goose faeces was more rapid than in sediments contaminated with dairy cow or deer faeces. Goose faeces also recorded a more rapid settling rate of faecal particles through the water column relative to dairy cow and deer faeces, suggesting a more efficient delivery of *E. coli* to streambed sediments associated with this faecal source. Our findings provide new evidence to improve understanding of the potential longer-term risks to both the environment and public health posed by sediments when contaminated with livestock, wildlife, and wildfowl faeces.

## 1. Introduction

Streambed sediments can harbour a range of terrestrially sourced pollutants, e.g., nutrients, plastics, heavy metals, faecal microbes, etc. [1,2]. Faecal contamination of the water environment following agricultural runoff and sewage overflow delivers faecal indicator organisms (FIOs; indicators of potential pathogen contamination) into suspension in river drainage networks, and, depending on river flow rates, cell-particle associations, and sedimentation rates, a proportion of FIOs will become entrained in streambed sediment [3]. The settling of faecal material and associated FIOs into the streambed sediment provides a potential legacy store of microbial pollution, which can result in further delayed impairment to water quality following resuspension. Legacy stores of pollutants remain in the environment for a protracted period (e.g., weeks, months, or years) beyond their first introduction. There is a growing body of research that has documented the delayed impacts that legacy phosphorus can have on water quality and associated management [4,5]), but legacy risks associated with environmental stores of FIOs require further investigation.

Recently there has been increased recognition and public awareness of the risks posed to water quality from sewage pollution and spills from combined sewer overflows (CSOs) [6,7]. However, those debates have focused on the immediate impacts on the hygienic status of the receiving water, but a secondary issue is that faecal pollution will contribute FIOs to the streambed environment, too, where they may be stored for longer periods in the sediment [8,9]. Contaminated streambed sediments downstream of CSOs represent an easily identifiable hotspot of potential legacy FIO pollution because of their proximity to point source discharges. By contrast, diffuse agricultural pollution to surface waters represents a much more challenging delivery of FIOs to identify and manage. This is because there is no single identifiable source in the landscape, and, therefore, the loading of stream and river sediments with FIOs from agricultural practices, although potentially not as intense as a sewage discharge spill, may be more chronic and represent a long-term hindrance to effectively managing microbial water quality.

In addition to agricultural and sewage sources, FIO loading of watercourses and streambed sediments can also originate from wildlife and wildfowl. Populations of deer, geese, and other wildlife may defecate directly into an aquatic environment, or their faecal depositions to land can be disrupted following rainfall events, with a proportion of FIOs subsequently mobilised and transferred to receiving waters [10]. Studies have reported on black-tailed deer and Canadian geese as contributors of FIOs in environmental matrices [11,12], and there are reports of increased FIO inputs to sediment stores of aquatic environments in catchments that can result from wildlife activity [13]. While the environmental persistence of sewage- and livestock-derived FIOs has been well studied, with key factors recognised as being influential in promoting or hindering survival, less is known about FIOs contributed by wildlife and wildfowl or whether there are any important differences in their survival characteristics. Temperature is recognised as one of the most important environmental variables that controls FIO persistence once excreted from the human or livestock gut environment into the wider landscape [14,15]; however, in comparison, empirical data and the associated evidence base of how wildlife- and wildfowl-derived FIOs respond to different temperatures when associated with a range of environmental matrices is limited. With respect to streambed sediment survival, *E. coli* from goose, deer, and bovine faeces introduced into sediments versus survival of indigenous strains was studied by Kiefer et al. [16] but only at ambient temperatures. Final concentrations of *E. coli* across all faecal types were comparable after 32 days; however, *E. coli* die-off rates in sediments contaminated by different faecal sources were variable. Smith et al. [14] evaluated the effect of temperatures oscillations from 17 °C to 28 °C, typical of a diurnal summer temperature range for the location of study (Maryland, USA) on populations of *E. coli* and enterococci in sediments and in the water column. Again, lower temperature regimes were not considered. Both of these studies simulated stream conditions using a flow chamber, which provided a steady stream of water above the sediment via a closed-circuit water reservoir.

The persistence of FIOs in bed sediments has been linked to other factors, such as availability of nutrients [17], sediment characteristics [14,18], and protection from ultraviolet radiation and predative organisms [19]. FIOs in streambed sediment can also regrow under some favourable conditions [20,21], thus providing a potentially long-term input into the overlying water column [22]. Association of FIOs with organic and/or mineral matter can play an important role in their delivery to streambed sediment relative to freely suspended cells because of the impact on increased settling speeds [23]. Extracellular polymeric substances of bacteria, important proteins that play a major role in cell–sediment flocculation, can aid delivery of bacteria to the bottom sediment because of an increase in the downward flux associated with higher floc mass [24]. The differential settling rates of faecal material associated with varying faecal types through the water column is, therefore, another factor that can influence the magnitude of FIOs stored within the streambed sediment, although there are little data available that report on rates of faecal sedimentation and how they vary among different sources, e.g., livestock, wildlife, or wildfowl, and their associated differences in faecal characteristics.

It is, therefore, important to study the survival pattern of *E. coli* from different faecal types, beyond the well-recognised human and livestock sources, at different temperatures to improve our knowledge on the persistence of *E. coli* in streambed sediment. The overarching aim of this study was to determine the persistence of *E. coli* derived from dairy cow, deer, and goose faecal sources introduced to streambed sediment mesocosms under different temperature regimes. The specific objectives of the experiment were to: (i) determine how die-off rates of *E. coli* vary in sediment contaminated with dairy cow, deer, and goose faeces; (ii) quantify how temperature influences concentrations of *E. coli* in faeces-contaminated streambed sediments; and (iii) evaluate how the solid constituents of dairy cow, deer, and goose faeces vary with respect to their settling rate once delivered to an aquatic environment.

## 2. Materials and Methods

A controlled laboratory experiment was carried out to determine the persistence of *E. coli* in streambed sediment under two constant temperature regimes (4 °C and 18 °C), which represent UK streambed temperatures experienced during very cold and very warm days in January (winter) and July (summer), respectively [25]. All experiments were carried out using incubators. Sampling was undertaken to monitor the difference in the persistence of *E. coli* derived from different common rural faecal sources (dairy cow, red deer, and greylag goose) once integrated into streambed sediment. Sediment samples were analysed more frequently in the early stages of the experiment, and sampling continued for up to 22 days to provide an overview of longer-term survival dynamics relative to the more rapid decline of FIOs that is commonly reported in aquatic environments [26].

### 2.1. Provenance of Faeces Used in All Experiments

All faeces were collected fresh for use in experiments, and the provenance of all faecal sources and sample collection is detailed in full in Afolabi et al. [10]. Briefly, fresh dairy faeces were collected from the livestock housing of a conventional dairy farm in Stirlingshire, Scotland. Fresh faeces of red deer were collected from the Scottish Deer Centre, Fife, Scotland. Fresh faeces from greylag geese were collected from the Royal Society for Protection of Birds (RSPB) reservation located on the shores of Loch Leven, Fife. After collection, all faeces were transferred immediately (<1 h) to the laboratory for use in the experiment, and, thus, no interim storage was required.

### 2.2. Artificial Sterile River Water Preparation

A standardised river water (soft water) was formulated from three stock solutions, which were prepared in advance following the method described by Smith et al. [27]. Stock 1 was composed of MgCl_2_·6H_2_O, 12.168 g/L, (0.06 mM), CaCl_2_·2H_2_O, 11.76 g/L (0.08 mM), and Ca(NO_3_)_2_·4H_2_O, 3.542 g/L (0.015 mM). Stock 2 was composed of CaCO_3_, 0.01872 g/L (0.170 mM), while Stock 3 was composed of Na_2_SO_4_, 16.334 g/L (0.115 mM), K_2_CO_3_, 1.725 g/L (0.0125 mM), and Na_2_CO_3_, 1.06 g/L (0.01 mM). All stock solutions were prepared in mg L^−1^ and vigorously stirred throughout the preparation, and sub samples of the final matrix were taken to verify the actual final concentration of cations and anions in the solution. Concentrations of major ions were determined by ion chromatography using a Dionex™ Aquion™ Ion Chromatography (IC) System (ThermoFisher, San Jose, CA, USA). A total of 2727 mL of Stock 2 was added to a 5-litre beaker, while 3 mL each of Stock 1 and Stock 3 were added to the beaker, and the solution was vigorously stirred to ensure that the solutes completely dissolved with final pH of 8.41. The artificial river water was sterilised in Duran bottles using an autoclave (15 min at 121 °C).

### 2.3. Preparation of Streambed Sediment

The streambed sediment was sourced from a local first-order agricultural stream and transported to the laboratory in a sterile polyethene bag. Approximately 5 kg of wet weight sediment was sterilised in an autoclave at 121 °C for 15 min to remove background microorganisms from the sediment. The sediment was then distributed into three clean foil trays with surface area of 324 cm^2^ each and oven-dried at temperature 100 °C for 72 h until the moisture content was completely removed, and sediment measurements recorded constant mass. The sediment was allowed to cool to room temperature and sieved using a sterile 2 mm sand sieve to remove debris, stones, and large particles before the preparation of mesocosms. The absence of opportunistic faecal indicator microbes (*E. coli* and enterococci) in the sterilised sediment was confirmed by streaking a suspension of sediment onto Membrane Lactose Glucuronide Agar (MLGA) (CM1031, Oxoid, Basingstoke, UK) and Slanetz and Bartley medium and recording zero growth after incubation.

### 2.4. Preparation of Mesocosms

Each treatment consisted of four replicates of a faecal/sediment mix per sampling day that were destructively harvested. The treatments were prepared as a mix of sterile sediment (dry) and fresh faeces at a weight ratio of 8:2, respectively. This mimicked sediment contamination, but clearly the ratio of faecal contamination to sediment can vary under environmental conditions. The sediment and faecal mix were homogenised in a sterile tray to ensure even distribution of cells. A total of 15 g of this contaminated mix was added to each 50 mL centrifuge tube (4 replicates per time point, 6 time points), and the tube were tapped to allow the sediment to settle evenly. Next, 30 mL of sterile artificial river water was slowly pipetted down the side of each tube to prevent agitation of the contaminated mix. The river water delivered moisture to the faecal/sediment mix. The overlying water was not flowing and, thus, provided a standing water scenario. The tubes were randomly divided into each treatment and arranged in plastic racks and stored in incubators at either 18 °C or 4 °C for 22 days. A destructive sampling approach was used whereby each treatment was sampled on Day 0, 2, 6, 9, 15, and 22 to monitor the persistence of *E. coli* in streambed sediment. Therefore, 72 mesocosms were used in total to allow for 4 replicates in each of the 3 treatments over 6 sampling days. Samples were collected more frequently in the early stages of the experiment to capture the more dynamic phase of population change.

### 2.5. Analysis of Streambed Sediment Particle Texture

The streambed sediment was analysed using a Coulter counter (Beckman Coulter L5230; Beckman Coulter (UK) Ltd., High Wycombe, UK). A sub-sample of the oven-dried, well-mixed and sieved streambed sediment was divided into three replicates. To accomplish this, 50 mL plastic sample bottles were filled with sediment to a depth of approximately 0.5 cm and topped up to 1.5 cm with distilled water. Next, 2 mL of dispersant sodium hexametaphosphate (Calgon) was added to the mixture to aid deflocculation. Then, the samples were agitated using a table shaker overnight to ensure homogeneity of the mixture. The samples were then prepared for analysis by stirring the mixtures using a magnetic stirrer for a minimum of 30 min, and the samples were run through Coulter counter machine to determine particle size distribution. The particle size composition for the sediment used in this experiment was categorised into clay (<0.002 mm), silt (0.002–0.059 mm), fine sand (0.06–0.19 mm), medium sand (0.2–0.59 mm), and coarse sand (0.6–2.0 mm), with the percent composition determined to be 13.80, 70.54, 13.73, 1.93, and 0%, respectively.

### 2.6. E. coli Enumeration in Streambed Sediment

On sampling days, approximately 3 g of contaminated mix (faecal streambed sediment) was randomly sampled from all replicates of each treatment using a sterile spatula after the removal of the overlying water using a pipette. To enumerate the *E. coli* present in the sediment, each 3 g sample was transferred to 27 mL of sterile river water in a 50 mL centrifuge tube and vortex-mixed for 30 s to ensure homogeneity prior to subsequent 1:10 serial dilution in PBS. Subsequently, 1 mL of each serially diluted sample was pipetted on to a 0.45 µm cellulose acetate membrane and washed through a vacuum-filtration unit (Sartorius Stedim Biotech., Goettingen, Germany) with ~20 mL of sterile PBS to ensure the capture of between 20 and 200 colony-forming units (CFU). To determine presumptive *E. coli*, the membranes were aseptically transferred to a Petri dish containing MLGA (CM1031, Oxoid, Basingstoke, UK), inverted, and incubated at 37 °C (±0.2 °C) for 18–24 h. The remaining sediment (~12 g) was used to determine gravimetric water content by drying at 100 °C for 48 h.

### 2.7. Rate of Faecal Material Sedimentation in Water

An experiment to infer the rate of sedimentation of faecal material delivered to water was conducted to complement the investigation of *E. coli* persistence in streambed sediment. Briefly, 10 g of faecal matter (dairy cow, red deer, and greylag goose) was weighed into a 50 mL centrifuge tube in replicate (*n* = 3) and 30 mL of distilled water added. The mixture was vigorously shaken until the faecal matter disaggregated in the water, and the tubes were then left to stand as the faecal material settled. The rate of sedimentation was inferred by measuring the change in water turbidity at 0, 1, 2, 3, 4, 5, 10, 20, 30, 60, 120, 180, 240, 300, 360, 420, 480, 540, 600, 660, and 720 min, with each replicate tube destructively sampled. Approximately 1 mL of the mixture was sampled at each time point and diluted with 9 mL of distilled water in a cuvette, and the sample was shaken to mix. All samples were then analysed for turbidity using a Hannah LP2000 benchtop turbidity metre (Hanna Instruments, Bedfordshire, UK).

### 2.8. Statistical Analysis

Statistical analysis was performed using Minitab (Minitab 18.0 software, Minitab Inc.: State College, PA, USA). Plate counts of *E. coli* were normalised by transforming to log_10_ CFU g^−1^ dry weight sediment. Analysis of variance (ANOVA) was used to test for differences in dry matter content and initial concentrations of *E. coli* in faeces and to test for differences in turbidity associated with the sedimentation experiment. Linear regression was used to estimate the rate of *E. coli* decline (*k*) in the streambed sediment. If any treatment recorded an initial period of growth, the linear model was fitted once the *E. coli* population began to decline (i.e., from the timepoint of peak concentration). Two-way ANOVA was used to test for differences in *E*. *coli* die-off characteristics (e.g., *k* values) in response to the effect of the treatments and the interaction between factors, and Tukey’s test was used for mean comparisons. Differences at the *p* < 0.05 level were considered statistically significant. *D*-values, which represent decimal reduction times, were calculated on the basis of the average rate of decline of *E. coli* following a log-linear die-off profile.

## 3. Results

### 3.1. Persistence of E. coli in Streambed Sediments

**Figure 1:**
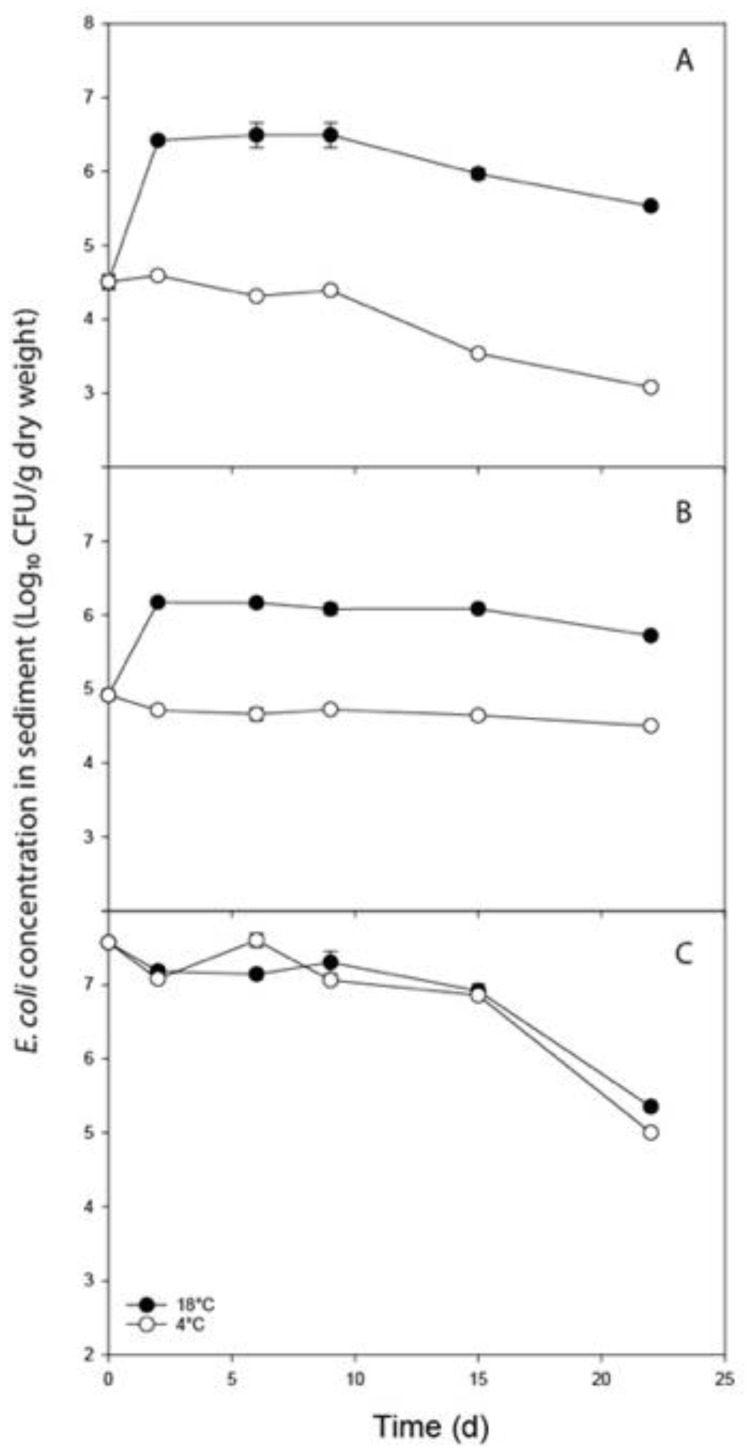
Persistence profiles in streambed sediment of *E. coli* sourced from (**A**) dairy cow, (**B**) red deer, and (**C**) greylag goose faeces. Data points are the mean of four replicates ± standard error.

The initial concentration of *E. coli* associated with fresh dairy cow, deer, and goose faeces prior to their mixing with sediment was 6.03, 6.03, and 8.24 log_10_ CFU g^−1^ dry weight, respectively. There was a significant difference in the starting concentrations of *E. coli* in fresh faeces (*p* < 0.001); goose faeces recorded the highest *E. coli* concentration, several orders of magnitude greater than concentrations in dairy and deer faeces. The dry matter content of fresh faeces from fresh dairy cows, red deer, and greylag geese was 70.02, 75.90, and 69.78%, respectively. On Day 0, concentrations of *E. coli* in the sediment following faecal contamination were also significantly different (*p* < 0.001). Sediment contaminated with goose faeces recorded the highest *E. coli* concentration, several orders of magnitude greater than concentrations in sediment contaminated with dairy and deer faeces (Figure 1). Concentrations of *E. coli* in sediment contaminated with dairy cow faeces were also significantly lower than concentrations recorded in sediment contaminated with deer faeces, reflecting the different dry matter contents of the faeces.

*E. coli* from dairy and deer faecal matter exposed to 18 °C exhibited initial *E. coli* growth followed by a slow decline to the end of the experiment but remained at levels far greater than the initial population of *E. coli* recorded on Day 0 (Figure 1). By contrast, concentrations of *E. coli* in sediment contaminated with goose faeces and held at 18 °C showed no evidence of an initial growth period, instead declining in population size from Day 0 and reaching 0.6 % of the initial population by Day 22 (cf. 1000% and 640% of initial population for sediments contaminated with dairy cow and deer faeces, respectively). The changes in percent survival of the initial *E. coli* population at 4 °C were less distinct, with sediments contaminated with all three faecal sources showing a degree of fluctuation up to Day 9, after which patterns of persistence diverged (Figure 1). At 4 °C, the proportion of the initial *E. coli* population remaining after 22 days in the sediment contaminated with goose, dairy, and deer faeces were each separated by an order of magnitude, with 0.3%, 4%, and 38% remaining, respectively.

A significant interaction occurred between temperature and faecal source (*p* < 0.001); higher concentrations of *E. coli* were observed in sediment contaminated with dairy cow and deer faeces at 18 °C relative to 4 °C, whereas sediments contaminated with goose faeces showed no distinction in *E. coli* concentrations between temperature treatments over the period of study (Figure 1). Linear regression was performed on the decline phase of the persistence profiles to model the die-off of *E. coli* across the different sediment treatments (Table 1). For those treatments that experienced growth, linear regression was initiated once the *E. coli* population began to decrease. Modelled decay constants were lowest for deer faeces and highest for goose faeces, with all three faecal types supporting significantly different rates of decline (*p* < 0.001; Table 1). The time needed for a decimal reduction in concentration (i.e., 1 log_10_ drop) ranged from 10 days, for sediment contaminated with goose faeces held at 4 °C, to 68.3 days, for sediment contaminated with deer faeces held at 18 °C. Sediment contaminated with dairy cow faeces recorded the greatest difference in *D*-values between the two temperature treatments (Table 1). The R^2^ values for *E. coli* die-off recorded in sediments contaminated with deer faeces indicated higher variability in their pattern of log-linear decay and, therefore, greater uncertainty associated with die-off parameters for the sediment contaminated with deer faeces.

### 3.2. Sediment and Sedimentation Rate Data

Silt dominated the sediment composition (70.6%), with clay and fine sand representing the other main constituents, albeit at much lower proportions (13.8% and 13.7%, respectively). The faecal material from the three faecal sources was artificially mixed with this sediment. To understand better how faecal material would dissipate through the water column and accumulate in the streambed sediment, an additional experiment was conducted to infer the rate of sedimentation of faecal material delivered to water.

**Figure 2:**
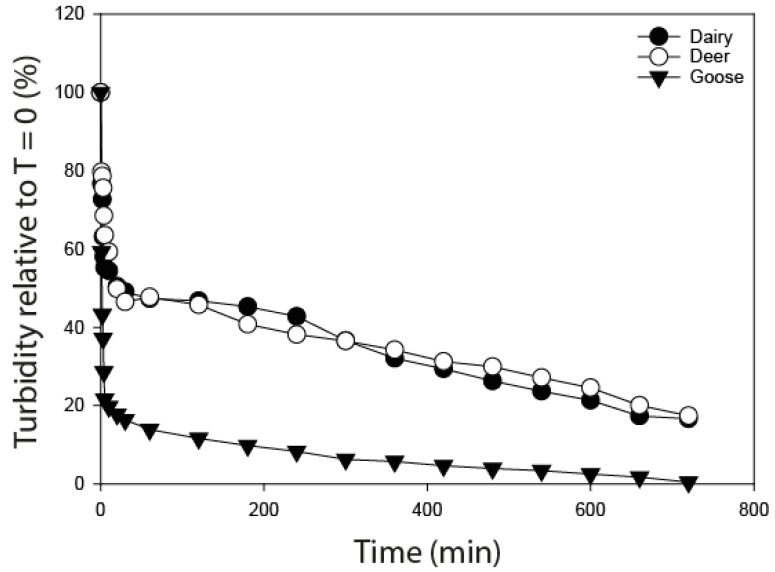
Sedimentation rate as measured by percentage change in turbidity over time.

A significantly lower starting turbidity was associated with the goose faeces treatment relative to the dairy cow and deer faeces (*p* < 0.01; Table 2). Normalising the turbidity data to percentage changes over time relative to the starting turbidity, therefore, provides a more meaningful visualisation of how patterns of sedimentation linked to the three faecal types differ (Figure 2). Patterns of sedimentation of the faecal constituents from dairy and deer faeces match very closely to each other. By contrast, goose faeces were observed to record much more rapid sedimentation times, reducing to ~20% of the original turbidity within 10 min, whereas a similar reduction in turbidity for dairy cow and deer faeces required more than 600 and 660 min, respectively. Despite the lower starting turbidity associated with goose faeces, the rate of change in the clarity of the water was also evidenced by the more rapid changes in recorded NTU values; the goose faeces treatment dropped by a magnitude of 3833 NTU between 0 and 2 min, with dairy and deer faeces recording a drop of 2543 NTU and 2116 NTU, respectively, over the same time period.

## 4. Discussion

Legacy stores of faecal pollution in streambed sediments can result in further delayed impacts on environmental quality and human health if resuspended into the overlying water column [28]. Characterising how different sources of faecal pollution can contribute to the legacy store of FIOs is, therefore, important for improved targeting of management advice and mitigation. Farming and wastewater treatment can be key contributors to faecal pollution, but there is recognition that wildlife and wildfowl activity in catchments can link to elevated FIO concentrations, a proportion of which will settle and persist in sediment stores [29]. This study provides new evidence to improve understanding of the potential risks posed by sediments when contaminated with wildlife and wildfowl faeces. The persistence patterns of *E. coli* in streambed sediment were found to vary as a function of faecal source and temperature. Fresh goose faeces accommodated the highest concentrations of *E. coli*; however, this faecal source also experienced the largest drop in concentration over the experiment duration, which also reflected the most rapid die-off rate relative to deer and dairy cow faeces. Temperature influenced patterns of survival; the warmer treatment was associated with regrowth of *E. coli* in sediments contaminated with both deer and dairy cow faeces. This led to distinctly different concentrations of *E. coli* over time supported at 18 °C versus 4 °C for these faecal types, but the temperature-driven response was not mirrored in sediments contaminated with goose-derived *E. coli*. The goose faeces also differed from dairy cow and deer faeces with respect to the recorded speed of settling of faecal particles in a water column, suggesting a more efficient delivery of *E. coli* to streambed sediments would occur when faecal material from geese enters a waterbody.

Differences in *E. coli* concentration in fresh faeces excreted by livestock, wildlife, and wildfowl are not unexpected, and studies have reported variability in *E. coli* shedding across different sources [30]. Differences in the initial concentration of *E. coli* likely reflect the diet associated with deer, dairy cows, and geese, and they also reflect the digestive tract characteristics and likely the cross-contamination from exposure to other animals in their habitats [31,32]. The dairy cow and deer faeces used in our study were collected from a working dairy farm and a deer park, respectively, where the animals were exposed to formulated feeds in addition to pasture. By contrast, the greylag goose population included a migratory and resident population that are much more free-roaming and largely unexposed to a managed diet, and the high initial concentration of *E. coli* in goose faeces was consistent with previous studies [11,33]. Experiments that use faeces as a natural carrier of indigenous FIOs to contaminate environmental matrices and then compare FIO survival responses across treatments provide an alternative to experiments that inoculate a known quantity of cells to a range of treatments. The former can make the assessment of the subsequent survival patterns more challenging because of uncertainties in how variation in starting concentrations of cell numbers may be propagated through the survival response, but such an approach is more reflective of real-world scenarios, and different experimental approaches offer different types of insight, provided that strengths and limitations are recognised [34].

The physical integrity of the different faecal matrices was lost through their combining with the streambed sediment. It is, therefore, difficult to suggest that differences in physical structure of the faeces played a role in determining the persistence patterns; however, the nature of the particles the faeces contain would differ and these would persist when combined with sediment. As discussed, the starting concentrations did differ and perhaps this was responsible for the more rapid *E. coli* die-off in the sediments contaminated with goose faeces, which after 22 days reached a concentration equivalent to the starting concentration of the sediments contaminated with deer and dairy cow faeces. There is evidence to suggest that experiments that use higher starting concentrations of FIOs are likely to record more obvious die-off than those experiments that use lower starting concentrations [35]. An alternative approach would have been to mix the sediment with different volumes of faeces to ensure the same FIO loading across all treatments; however, doing so would result in different ratios of the faeces: sediment mix, which itself could influence the survival response of the FIOs because of differences in nutrient supply to the bacteria [36].

The survival and growth of *E. coli* in the environment has been attributed to the influence of temperature [20]. In our study, the warmer (18 °C) temperature treatment supported higher concentrations of *E. coli* over time relative to the lower temperature treatment (4 °C). Despite 18 °C not being the optimal temperature to encourage *E. coli* growth, temperatures of a similar magnitude in combination with the protective matrix of a sediment environment have been reported to facilitate increases in *E. coli* population and support their survival [18,20]. For example, a two- to fourfold increase in *E. coli* concentration was recorded in faeces-contaminated sediment after a two-day incubation at 14 °C [18]. Temperature is recognised as an important factor that controls the survival of microorganisms in the environmental matrices, including enteric bacteria excreted from warm-blooded animals’ guts [37]. While warmer temperatures supported growth of *E. coli* in the sediment contaminated with dairy and deer faeces, no significant regrowth was recorded in *E. coli* held in sediment at 4 °C for all faecal sources. The lack of growth of *E. coli* in goose faeces may be attributed to strain and genotype differences of *E. coli* in the faecal matter [38], and potential differences in *E. coli* strains contained in the different faecal sources will likely exhibit variation in intrinsic survival capacities. How those strains respond to competition with natural bacterial communities can also influence the survival outcomes. The difference in *E. coli* response at different temperatures is clear when comparing each faecal treatment individually, e.g., the differences observed for temperature effects in dairy faeces and in deer faeces, because both temperature treatments for each respective faecal source started with the same *E. coli* concentration on Day 0. Although the influence of temperature itself is not a novel finding, the *E. coli* growth rate and magnitude of increase recorded for both faecal sources is still substantial and provides important evidence of how FIOs can increase in environmental matrices under varying conditions and provides information to support FIO fate and transfer modelling [34]. The lack of growth in all faeces-contaminated sediments at lower temperatures is likely due to a reduction in the metabolic process of *E. coli* in the environmental matrices [12]. Consequently, some cells may have experienced mechanical damage to their cell structure or entered a viable-but-non-culturable (VBNC) state [39], limiting opportunities for cell replication.

The inactivation rate of *E. coli* in the environment can be influenced by the sediment composition and sedimentation rate, which links to particle size and the organic matter content, although the role of different sediment characteristics in FIO survival is not straightforward and interacts with other environmental factors [16,18]. In this study, only one type of sediment was used because the focus of the research was to determine the influence of different faecal types spanning livestock, wildlife, and wildfowl sources combined with influences of temperature. An investigation into whether the persistence patterns recorded for this sediment composition hold across other sediment types dominated by sand or clay fractions would be important to further support the evidence base of how various wildlife and wildfowl sources of FIOs persist in the environment. This would help to refine risk assessments (e.g., [40,41]) of landscapes frequented by large wildlife and wildfowl populations by identifying the factors that combine to generate legacy FIO hotspots in stream and river networks.

Rates of FIO accrual in bottom sediments are governed by their attachment to particles [42]. Although some FIOs will enter a waterbody as freely suspended cells, a large proportion will be associated with mineral or organic particles. Those FIOs that have attached or remain associated with physical material will settle out into underlying bed sediment relatively faster than free floating FIOs [8], because the rate of settling of suspended particles depends on the mass of the particles. This study focused on quantifying persistence of FIOs over time in sediments contaminated with different faecal sources, but a secondary aim was to identify whether the different faecal sources would influence the rate of faecal material (and, by association, FIO) delivery to the streambed sediment. The constituent parts of goose faeces, when mixed with water, were found to settle at a more rapid rate than those associated with deer and dairy faeces. Diet again, as discussed earlier, likely influences the composition of the faecal material and will dictate, to some extent, how the faecal material disaggregates and settles through a water column [43]. All faecal types were fresh, but the goose faeces had marginally higher (<0.5% difference) and higher (~6% difference) moisture content than the dairy cow and deer faeces, respectively. This would suggest that the drier the faecal matter, the more likely the faecal particles are to remain in suspension in the water column for a longer duration. However, the results of this study cannot conclude whether dry weight or faecal type is the factor driving the rate of settling. Further research is needed to investigate the settling rates of different faecal types across a spectrum of recorded dry weights, which would also reflect different ages of faeces.

This study did not use flowing water chambers such as those used by [14,16]. Instead, the mesocosm design reflects a shallow water depth above the sediment; the lack of flow would have physical effects as well as impacts on oxygenation. Such a scenario may be more representative of areas where stagnant water may accumulate, such as in backwaters. However, such areas will hydrologically reconnect with a stream network during wet weather and provide opportunities for downstream pollution if sediment resuspension occurs. There were some other limitations in that no sediment/faecal chemistry was undertaken and microcosms were capped, which would have some influence on aeration/anaerobicity. Despite these potential limitations, findings in this study underscore the importance of warmer temperatures in promoting higher concentrations of *E. coli* in sediments contaminated with deer and dairy cow faeces, which are then likely to result in hotspots of potential legacy pollution.

## 5. Conclusions

Characterising how *E. coli* from different catchment sources survive in streambed sediment under varying temperature regimes can help catchment managers and environmental regulators understand the potential for faecal pollution and public health implications following resuspension of legacy FIO stores. In this study, we combine such data with the sedimentation rates of faeces contributed to water from dairy cows, deer, and geese to highlight the potential for differential loading of *E. coli* to the streambed environment when associated with a faecal source. The concept of delayed impairment of water quality from legacy phosphorus is well recognised, but, equally, other pollutants, such as FIOs, can accumulate in catchment stores and cause rainfall-independent water quality impacts if disturbed, e.g., by recreational water users or livestock activity in water courses and high flow impacts following sediment resuspension. The dynamic nature of FIO die-off means that these sediment hotspots may have time-limited risk periods that respond to temperature influences on survival. Further laboratory research and field quantification on survival of wildlife- and wildfowl-derived *E. coli* in different sediment types and catchment settings through contrasting seasons will deliver further evidence to support our knowledge and risk assessment of other non-agricultural and non-human FIO pollution sources in catchments.

## Figures and Tables

**Table 1:** Linear model parameter values for *E. coli* decay in sediment contaminated with different faecal types; superscript letters indicate grouping information for *k* using Tukey’s pairwise comparisons.

			Die-Off Phase Coefficients						
Treatment	Dairy Cow			Deer			Goose		
	*k* (day^−1^)	R^2^	*D*-value (days)	*k* (day^−1^)	R^2^	*D*-value (days)	*k* (day^−1^)	R^2^	*D*-value (days)
18 °C	0.105 ^C^	72.9	21.9	0.048 ^E^	68.3	48.0	0.193 ^A^	73.8	11.9
4 °C	0.164 ^D^	89.2	14.0	0.032 ^E^	53.3	72.0	0.230 ^B^	73.7	10.0

**Table 2:** Turbidity (Nephelometric Turbidity Units) values over time for each of the faecal treatments.

	Turbidity (Nephelometric Turbidity Units)					
	Dairy Cow Faeces		Deer Faeces		Goose Faeces	
Time (min)	Mean	SE	Mean	SE	Mean	SE
0	9316.7	455.7	9926.7	27.3	6756.7	636.1
1	7146.7	81.7	7916.7	38.4	4006.7	327.5
2	6773.3	271.7	7810.0	268.5	2923.3	86.7
3	5886.7	128.4	7506.7	146.2	2510.0	90.7
4	5406.7	69.8	6803.3	236.9	1933.3	167.6
5	5146.7	33.3	6310.0	17.3	1460.0	35.1
10	5073.3	82.1	5886.7	349.2	1333.3	16.7
20	4700.0	65.1	4943.3	58.4	1200.0	37.9
30	4570.0	63.5	4613.3	172.3	1103.3	21.9
60	4406.7	17.6	4736.7	12.0	936.7	31.8
120	4356.7	82.5	4540.0	105.4	783.3	46.7
180	4216.7	18.6	4046.7	92.4	660.0	10.0
240	3983.3	101.7	3786.7	34.8	560.0	28.9
300	3410.0	258.9	3623.3	60.6	422.5	11.8
360	2986.7	173.7	3396.7	59.0	384.9	5.9
420	2736.7	49.8	3100.0	61.1	311.5	7.1
480	2446.7	80.1	2970.0	35.1	263.9	15.7
540	2203.3	103.7	2690.0	70.0	228.3	21.2
600	1986.7	145.3	2436.7	78.0	168.8	10.5
660	1613.3	240.4	1990.0	236.9	116.5	10.7
720	1546.7	147.2	1726.7	312.1	29.5	6.5

## Data Availability

Please contact the authors to request a copy of the raw data.
